# pH-Responsive Bovine Serum Albumin Nanoparticles Encapsulating Doxorubicin-Based Complexes Induce Cuproptosis in Lung Cancer Cells

**DOI:** 10.3390/pharmaceutics18050526

**Published:** 2026-04-26

**Authors:** Haiying Zhang, Xuanjia Chen, Shihui Qiao, Huanfeng Meng, Hui Long, Huamin Zhong, Yiheng Liu, Yun Song, Yanan Gao, Yan Liu, Lujia Mao

**Affiliations:** 1 Hainan Provincial Key Laboratory of Research and Development on Tropical Herbs, Engineering Research Center of Tropical Medicine Innovation and Transformation of Ministry of Education, Key Laboratory of Tropical Translational Medicine of Ministry of Education, Haikou Key Laboratory of Li Nationality Medicine, College of Pharmacy/College of Basic Medical Sciences, Hainan Academy of Medical Science, Hainan Medical University, Haikou 571199, China; zhanghaiying@muhn.edu.cn (H.Z.);; 2 Biobank, Hainan Medical University, Haikou 571199, China; 3 Affiliated Haikou Hospital of Xiangya Medical College, Central South University, Haikou 570208, China

**Keywords:** cuproptosis, pH-responsive, multidrug delivery system, doxorubicin, bortezomib

## Abstract

**Background/Objectives**: This study investigates the induction of cuproptosis in A549 lung cancer cells by doxorubicin (DOX) complexes and the development of pH-responsive bovine serum albumin (BSA)-based nanocarriers for their delivery. We successfully synthesized and characterized two novel complexes: DOX–Cu, where DOX acts as a ligand for Cu(II), and DOX–BTZ, a conjugate formed between DOX and the proteasome inhibitor bortezomib (BTZ). **Methods**: Spectroscopic and NMR analyses were performed to confirm the formation of the complexes. In vitro assays were conducted to evaluate cytotoxicity in A549 cells, alongside assessment of DLAT aggregation as a marker of cuproptosis. The formulation of DOX into BSA nanoparticles (DOX–Cu@BSA NPs and DOX–BTZ@BSA NPs) was carried out to evaluate potential alleviation of DOX-induced cytotoxicity in cardiomyocytes in vitro. Fluorescence quenching and molecular docking studies were employed to investigate the binding interactions between the complexes and BSA. Cellular uptake experiments were performed to assess nanoparticle internalization into A549 cells. **Results**: Both complexes exhibited superior cytotoxicity against A549 cells compared to individual components. This enhanced cell death was associated with significant aggregation of dihydrolipoamide S-acetyltransferase (DLAT), a key marker of cuproptosis, suggesting the involvement of this copper-dependent cell death pathway. The BSA nanoparticles displayed favorable characteristics, including uniform size (~190 nm), high encapsulation efficiency (~75–79%), and colloidal stability. Crucially, they exhibited a pH-responsive drug release profile, with significantly accelerated release under acidic conditions (pH 5.7) mimicking the tumor microenvironment. Fluorescence quenching and molecular docking studies revealed strong, spontaneous binding between the complexes and BSA, primarily driven by hydrophobic interactions. Cellular uptake experiments confirmed efficient internalization of the nanoparticles into A549 cells. **Conclusions**: Collectively, this work offers a proof-of-concept for a strategy of utilizing BSA-based multidrug delivery systems for cuproptosis induction, offering a potential avenue to enhance therapeutic efficacy while reducing systemic toxicity in lung cancer treatment.

## 1. Introduction

**Figure 1:**
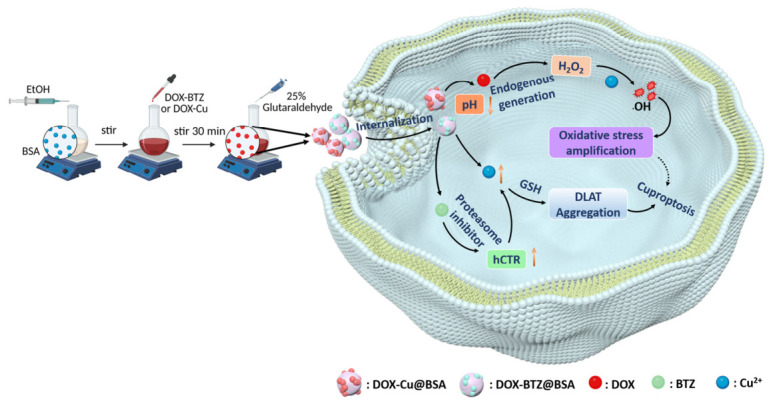
Illustrating the preparation of the nanoparticles and the role of DOX-based complexes in enhancing cuproptosis therapy.

Copper, an essential trace element and redox-active transition metal, plays a critical role in maintaining normal physiological functions through its involvement in enzymatic activity and transcriptional regulation [1]. However, copper homeostasis must be tightly regulated, as both deficiency and excess can lead to cytotoxic effects. Mounting evidence indicates that copper levels are significantly elevated in malignant tissues and serum samples from cancer patients compared to healthy individuals, spurring growing interest in its pathophysiological roles [2]. In particular, copper-dependent regulatory mechanisms and the recently identified form of copper-mediated cell death, known as cuproptosis, have become major focuses in cancer research [3]. Distinct from classical apoptosis or necrosis, copper-induced cell death has attracted considerable attention due to its potential for therapeutic exploitation, especially in overcoming resistance to conventional chemotherapy [4]. Preclinical studies increasingly suggest that targeted manipulation of copper metabolism or direct copper-based treatments can inhibit tumor growth, offering a promising strategy for addressing chemoresistant cancers and transforming oncology treatment frameworks [5]. Thus, it is hypothesized that combining exogenous Cu(II) delivery with hypoxia alleviation could robustly enhance cuproptosis in anticancer therapy (Figure 1).

**Figure 2:**
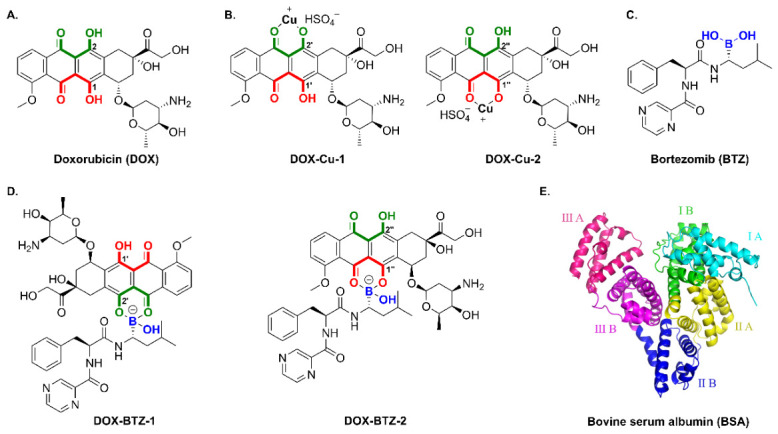
Crystal and molecular structure. (**A**) DOX, (**B**) DOX–Cu, (**C**) BTZ, (**D**) DOX–BTZ and (**E**) BSA.

Doxorubicin (DOX, Figure 2A), a clinically important chemotherapeutic agent, demonstrates potent activity against a broad spectrum of solid tumors [6]. It potentiates the production of endogenous hydrogen peroxide (H_2_O_2_) through a sequential catalytic process mediated by nicotinamide adenine dinucleotide phosphate oxidase 4 (NOX4) [7]. The resulting H_2_O_2_ then reacts with Cu(II) to generate hydroxyl radicals (•OH), which amplify oxidative stress, and molecular oxygen (O_2_), which helps alleviate hypoxia [5]. Under physiological conditions, the β-hydroxy ketone moiety in DOX exhibits strong Cu(II)-chelating ability (Figure 2B), enabling it to act as an effective ligand for Cu(II) [8]. Thus, the strategic incorporation of Cu(II) offers a proof-of-concept for a strategy to enhance the antitumor efficacy of DOX-based treatments.

Bortezomib (BTZ, Figure 2C), a boronic acid-based dipeptide proteasome inhibitor, selectively inhibits the chymotrypsin-like activity of the mammalian 20S proteasome [9]. By impairing proteasomal degradation, BTZ stabilizes the human copper transporter 1 (hCTR1), leading to enhanced intracellular accumulation of Cu(II) and subsequent copper-dependent cytotoxicity [10]. Despite this mechanistic insight, BTZ shows limited clinical efficacy against solid tumors, largely attributable to its sub-optimal pharmacokinetics including poor tissue penetration and rapid clearance [11]. Notably, combination therapies incorporating BTZ and DOX have demonstrated synergistic antitumor effects in preclinical models, surpassing monotherapy outcomes through complementary actions: (1) DOX-induced oxidative stress potentiates copper-mediated DNA damage, while (2) BTZ-mediated proteasome inhibition suppresses pro-survival autophagy and chemoresistance pathways [12]. Structural analyses confirm the spontaneous self-assembly of a tetraborate-bridged DOX–BTZ conjugate (Figure 2D), formed via covalent bonding between the boronic acid group of BTZ and the β-hydroxy ketone moiety of DOX, stabilized by Lewis acid–base interactions. This hybrid compound represents a novel metallo-chemotherapeutic platform with the potential to overcome conventional treatment limitations [13,14,15,16,17].

Albumin amplifies macropinocytosis-driven catabolic processes in malignant cells, while simultaneously functioning as a highly effective nanocarrier for targeted anticancer therapeutic delivery [18]. Serum albumin (SA), the most abundant plasma protein in vertebrates, plays a central role in transporting a wide range of endogenous and exogenous molecules [19]. Among protein-based carriers, bovine serum albumin (BSA, Figure 2E) offers distinct advantages, including non-toxicity, biodegradability, biocompatibility, high drug-binding affinity, and suitability for injection [20,21]. Although studies have reported DOX–Cu complexes [22,23], combination strategies of DOX with BTZ [12], and albumin drug delivery systems [18,19,20], a pH-responsive multidrug delivery system designed for specific induction of cuproptosis and synergistic use with proteasome inhibitors to overcome drug resistance has not yet been systematically documented. In this study, we conducted: (1) spectroscopic and molecular docking studies confirming the binding between BSA and DOX–Cu/DOX–BTZ complexes; (2) comprehensively evaluates both DOX–Cu and DOX–BTZ complexes for their ability to trigger DLAT aggregation, a hallmark of cuproptosis; and (3) demonstrates the successful encapsulation of these specific cuproptosis-inducing complexes into a pH-responsive BSA nanocarrier system [24].

## 2. Materials and Methods

### 2.1. Chemical and Reagents

Doxorubicin (Cat. No. E080136), bortezomib (Cat. No. E120421), bovine serum albumin (Cat. No. E080147), 25% glutaraldehyde (Cat. No. B2328238), ethanol (Cat. No. W320322), dimethyl sulfoxide-*d*_6_ (DMSO-*d*_6_, Cat. No. E090043), copper(II) chloride dihydrate (Cat. No. A43331), sodium phosphate dihydrate (Cat. No. EB000174), copper sulfate pentahydrate (Cat. No. M0113828), and disodium hydrogen phosphate dodecahydrate (Cat. No. A16405) were obtained from Energy Chemical Co., Ltd. (Shanghai, China) and used without further purification. Dulbecco’s Modified Eagle Medium (DMEM, Cat. No. D6429) and fetal bovine serum (FBS, Cat. No. F0193) were purchased from Sigma (Shanghai, China). Trypsin–EDTA (0.25%, Cat. No. 25200056) was acquired from Gibco (Shanghai, China). Penicillin–streptomycin solution (100×) was procured from Pricella (Wuhan, China). Phosphate-buffered saline (PBS, Cat. No. BL302A) was obtained from Biosharp (Beijing, China). The Cell Counting Kit-8 (CCK-8) assay kit (Cat. No. C0039) was purchased from Beyotime (Shanghai, China). Z-VAD(OH)-FMK (Z-VAD, Cat. No. HY-16658B), Necrostatin-1 (Nec-1, Cat. No. HY-15760) were purchased from MedChemExpress (Shanghai, China). Ammonium tetrathiomolybdate (TTM, Cat. No. A828261) was purchased from Macklin (Shanghai, China). The A549 cell line (Cat. No. CL-0016) was procured from Procell Life Science & Technology (Wuhan, China), stored at Shanghai Origincell Biological Cryo Equipment Co., Ltd. R&D. Institute (Shanghai, China), and the AC16 cell line (Cat. No. STCC13101P) was obtained from Chinese Servicebio (Wuhan, China). All other chemicals were of analytical grade.

### 2.2. Sample Preparation

NaH_2_PO_4_·2H_2_O (31.2200 g) and Na_2_HPO_4_·12H_2_O (71.6280 g) were separately dissolved in deionized water and each diluted to a final volume of 1 L. A concentrated phosphate buffer stock solution (pH ~7.4) was subsequently prepared by mixing 19 mL of the NaH_2_PO_4_·2H_2_O solution with 81 mL of the Na_2_HPO_4_·12H_2_O solution. Finally, 75 mL of this stock solution was transferred to a 250 mL volumetric flask and diluted to the mark with deionized water to yield a 60 mM phosphate-buffered saline (PBS, pH 7.4).

Bovine serum albumin (BSA, 83.1 mg) at a final concentration of 1.25 mmol was dispersed in 60 mM sodium phosphate buffer (pH 7.4, 50 mL). Stock solutions of test compounds (0.5 mM) were prepared through dissolution of crystalline solids in a binary solvent system comprising ethanol and water, followed by sequential dilution to target experimental concentrations using the same phosphate buffer medium. All procedures strictly adhered to the protocol outlined in reference, ensuring consistency in buffer composition and solvent conditions across experimental groups [25].

The DOX–Cu complex was synthesized via stepwise introduction of DOX (2.9 mg, 1.0 equiv.) into an aqueous solution containing CuSO_4_·5H_2_O (1.2 mg, 1.0 equiv.) dissolved in 3.0 mL of deionized water. This mixture was subsequently subjected to magnetic stirring at 25 °C for 2 h to facilitate complex formation, which was recrystallized from the mixture of water and EtOH.

The DOX–BTZ coordination complex was prepared via sequential introduction of DOX (2.9 mg, 1.0 equiv.) into a binary solvent system comprising H_2_O (1 mL) and ethanol (2 mL) in a 1:2 volume ratio, pre-loaded with BTZ (1.9 mg, 1.0 equiv.). The reaction mixture was subjected to magnetic stirring at 25 °C for 2 h to facilitate complexation kinetics, which was purified via recrystallization.

### 2.3. NMR Measurements

All nuclear magnetic resonance (NMR) spectra were acquired at 25 °C using a JEOL JNM-ECZ400S/L spectrometer system (JEOL Ltd., Tokyo, Japan) equipped with dual-frequency capabilities (^1^H at 400 MHz; ^11^B at 128 MHz). Proton (^1^H) chemical shifts were referenced to tetramethylsilane (TMS) as the primary internal standard, with secondary calibration achieved through residual solute signals in the respective deuterated solvent medium—specifically, DMSO-*d*_6_ exhibiting a characteristic residual proton resonance at δ = 2.50 ppm. For ^11^B NMR acquisition, chemical shifts were determined relative to boron trifluoride diethyl ether complex (BF_3_·Et_2_O) utilized as an external standard, ensuring absolute chemical shift accuracy.

The DOX–BTZ conjugate was synthesized via stepwise addition of DOX (20.0 mg, 1.0 equiv.) to a deuterated solvent system containing DMSO-*d*_6_ (0.5 mL) pre-equilibrated with BTZ (13.3 mg, 1.0 equiv.). The reaction mixture was subjected to magnetic stirring at 25 °C for 2 h to ensure complete complexation.

### 2.4. UV Spectroscopy Measurements

Ultraviolet–visible (UV–Vis) absorption spectra were recorded using a PGeneral T6 spectrophotometer (Puxi General Instrument Co., Ltd., Beijing, China) across the spectral range of 190–800 nm at 25 °C.

### 2.5. FT-IR Measurements

Fourier transform infrared (FT-IR) spectroscopic analyses were conducted at 25 °C using a Thermo Fisher Scientific Nicolet iS5 spectrometer (Thermo Fisher Scientific, Waltham, MA, USA) equipped with a deuterated triglycine sulfate (DTGS) detector. Each spectrum was acquired through co-addition of 16 individual scans at a nominal resolution of 0.4 cm^−1^. The measurement range was set from 400 cm^−1^ to 4000 cm^−1^.

### 2.6. CCK-8 Assay

#### 2.6.1. A549 Cell Viability

A549 cells were seeded into 96-well plates at a density of 8000 cells per well and incubated overnight. Cell proliferation was assessed using the CCK-8 assay. After 48 h of treatment with DOX, BTZ, CuCl_2_·2H_2_O, DOX–Cu, or DOX–BTZ (each at 0.0625 μM), the supernatant was aspirated. The dosing concentration was determined based on preliminary dose–response experiments and literature reports [26]. The aim is to use sub-toxic ranges to evaluate synergy and cuproptosis induction without overwhelming cytotoxicity. Each well was then refilled with 100 μL of DMEM enriched with 10% fetal bovine serum (FBS) and 10% CCK-8 reagent (C0039, Beyotime Biotech Inc.), followed by a 37 °C incubation period lasting 90 min. Optical density measurements at 450 nm were acquired using a microplate spectrophotometer (BioTek Instruments Inc., Winooski, VT, USA). Data presentation adopted the format of mean ± standard error of the mean (SEM). All statistical computations were executed through GraphPad Prism software (version 9.5, USA). Prior to implementing one-way ANOVA, the assumption of variance homogeneity was tested; subsequent pairwise comparisons were applied when statistically justified. A threshold of *p* < 0.05 was established to denote statistically significant differences. All experiments were performed in six technical replicates per condition and repeated in six independent biological replicates (*n* = 6).

#### 2.6.2. Viability of AC16 Cells

AC16 human cardiomyocytes were cultured in Dulbecco’s Modified Eagle’s Medium (Sigma, Shanghai, China) containing 10% (*v*/*v*) Fetal Bovine Serum (Sigma, Shanghai, China) and 50 μg·mL^−1^ penicillin–streptomycin (Pricella). Cells were seeded into 96-well plates at a density of 5 × 10^3^ cells per well and incubated for 24 h. After treatment with the indicated reagents for 24 h, 10 μL of CCK-8 solution (Beyotime, Shanghai, China) was added to each well, and the plates were incubated at 37 °C for 0.5–1 h. The absorbance at 450 nm was measured using a microplate reader. Cell viability was normalized to the blank control group (set as 100%), and the viability of treated groups was expressed as a percentage relative to the control. All experiments were performed in six technical replicates per condition and repeated in six independent biological replicates (*n* = 6).

#### 2.6.3. Full Dose–Response Testing Protocol and Calculation of IC_50_ Values

A549 cells in the logarithmic growth phase were seeded into 96-well plates at a density of 8 × 10^3^ cells per well in 100 μL of DMEM medium supplemented with 10% fetal bovine serum (FBS) and incubated overnight at 37 °C with 5% CO_2_ to allow cell attachment. The medium was then replaced with fresh medium containing DOX–BTZ@BSA NPs or DOX–Cu@BSA NPs. Each compound was tested at six concentrations (0.03125, 0.0625, 0.125, 0.25, 0.5, and 1 μM), while the control group received an equal volume of drug-free medium. After incubation for 24 or 48 h, 10 μL of CCK-8 solution was added to each well, followed by further incubation at 37 °C for 1 h. The absorbance (OD) at 450 nm was measured using a microplate reader (BioTek Instruments Inc.). Data are presented as mean ± standard error of the mean (SEM). All statistical analyses were performed using GraphPad Prism software (version 9.5, USA). Homogeneity of variance was rigorously tested prior to one-way ANOVA; subsequent pairwise comparisons were conducted when statistically justified. A *p*-value < 0.05 was considered statistically significant. All experiments were performed in six technical replicates per condition and repeated in six independent biological replicates (*n* = 6).

#### 2.6.4. Cell Death Pathway Inhibitor Rescue Assay

A549 cells in the logarithmic growth phase were seeded into 96-well plates at a density of 8 × 10^4^ cells/well and incubated in DMEM medium supplemented with 10% FBS at 37 °C and 5% CO_2_ for 24 h to allow cell attachment. After removing the original culture medium, the experimental groups were treated with DOX–Cu@BSA NPs or DOX–BTZ@BSA NPs (both at a concentration of 0.0625 μM) either alone or in combination with Tetrathiomolybdate (TTM, 100 μM), Necrostatin-1 (Nec-1, 20 μM), or Z-VAD(OH)-FMK (Z-VAD, 20 μM), while the control group received an equal volume of drug-free medium. The incubation continued for 48 h. Subsequently, 10 μL of CCK-8 reagent was added to each well, followed by incubation in the dark for 1 h. The absorbance (OD) at 450 nm was measured using a microplate reader. Data were presented as mean ± standard error of the mean (SEM), and statistical analysis was performed using GraphPad Prism 9.5. After confirming homogeneity of variance, one-way ANOVA and pairwise comparisons were conducted. A *p*-value < 0.05 was considered statistically significant. All experiments were performed in triplicate technical replicates per condition and repeated in three independent biological replicates (*n* = 3).

### 2.7. Analysis of DLAT in A549 Cells

#### 2.7.1. DLAT Immunofluorescence Assay of DOX Complexes

A549 cells seeded on glass slides were treated for 24 h with PBS, CuCl_2_·2H_2_O, BTZ, DOX, DOX–BTZ, or DOX–Cu (each at 100 μg·mL^−1^). After treatment, the cells were fixed with 4% paraformaldehyde (PFA) for 15 min and permeabilized with 1% Triton X-100. Non-specific binding was blocked with 5% bovine serum albumin (BSA). The samples were then incubated overnight at 4 °C with primary antibodies against DLAT. After PBS washing, the cells were incubated with corresponding fluorescently labeled secondary antibodies for 1 h at 25 °C. Following another PBS wash, cell nuclei were stained with DAPI. Finally, the slides were mounted, and immunofluorescence images were captured using confocal (FV3000, Olympus Corporation, Tokyo, Japan) laser scanning microscopy (CLSM). All experiments were performed in triplicate technical replicates per condition and repeated in three independent biological replicates (*n* = 3).

#### 2.7.2. DLAT Immunofluorescence Assay of Nanoparticles

A549 cells were seeded into confocal dishes at a density of 5 × 10^4^ cells per well. After adherence, the cells were treated with either DOX–Cu@BSA NPs or DOX–BTZ@NPs (both at a concentration of 0.0625 μM) alone or in combination with TTM for 48 h. After continuing incubation for 48 h, the culture medium was discarded, and the cells were washed with PBS, fixed with 4% paraformaldehyde at 25 °C for 30 min, and permeabilized with 1% Triton X-100. Following blocking with 5% BSA for 1 h, sequential incubations were performed with primary antibodies (4 °C overnight), fluorescent secondary antibodies (25 °C, protected from light, 1 h), and DAPI nuclear staining (25 °C, 5 min). Finally, images were captured using CLSM. All experiments were performed in triplicate technical replicates per condition and repeated in three independent biological replicates (*n* = 3).

### 2.8. Fluorescence Spectroscopy

Fluorescence spectra were recorded on a Hitachi F-4700 spectrofluorometer (Hitachi High-Tech Corporation, Tokyo, Japan) equipped with a xenon lamp and a 10 mm quartz cuvette. Unless otherwise specified, the temperature was maintained at 25 °C throughout the experiments. Instrument parameters were optimized to ensure consistency and reproducibility. Fluorescence emission parameters were configured as follows: emission slit width set to 5 nm for optimal spectral resolution, with a scan rate of 1200 nm·min^−1^ to balance acquisition speed and signal fidelity. Excitation was precisely tuned to 280 nm, characteristic of aromatic amino acid absorption in protein fluorophores, to maximize signal detection efficiency. Emission spectra were collected from 290 to 450 nm for protein solutions, encompassing the primary fluorescence signatures of tryptophan (350 nm) and tyrosine residues. The inner filter effect, often observed at high analyte concentrations, reduces excitation energy or absorbs emitted fluorescence, thereby limiting optical pathways and attenuating signal intensity. To mitigate this issue, we optimized concentration ranges through preliminary calibration, ensuring accurate quantification without spectral distortions [27]. All datasets derive from triplicate independent measurements.

Three-dimensional fluorescence emission profiles were recorded for 5 μM BSA solutions under native conditions and following ligand binding at drug-to-protein molar ratios of 5:1 and 10:1. Spectra were obtained by increasing the excitation wavelength from 220 to 350 nm in 5 nm steps, while measuring the corresponding emission spectra across the 220–500 nm range.

This investigation examined drug-induced fluorescence quenching mechanisms in BSA using a controlled experimental framework. The BSA concentration was precisely maintained at 5.0 μM throughout all assays to ensure consistent protein environment, while drug concentrations were titrated across a 5–50 μM range in 5 μM increments to map dose–response relationships. Fluorescence emission spectra were acquired at three physiologically relevant temperatures (298 K, 303 K, and 310 K) using a thermos tatted cuvette holder to elucidate temperature-dependent quenching kinetics and thermodynamic parameters. All experiments were performed under controlled conditions to ensure reproducibility.

The underlying quenching mechanism was investigated by analyzing the fluorescence data using the Stern–Volmer equation [28]:

(1) $$F_{0}/F=1+K_{SV}\left[Q\right]=1+k_{q}\tau_{0}[Q]$$

here, *F*_0_ signifies the fluorescence intensity of BSA when no drugs are present, whereas *F* indicates the intensity in the presence of the drugs. In this context, [*Q*] denotes the concentration of the drug, while *K*_SV_ represents the Stern–Volmer quenching constant being utilized.

The *k*_q_ denotes the bimolecular quenching rate constant

(2) $$k_{q}=K_{SV}/\tau_{0}$$

where *τ*_0_ is the average lifetime of BSA, which was taken as 6 × 10^−9^ s for BSA [28].

The intrinsic fluorescence quenching of bovine serum albumin (BSA) induced by drug binding arises from the formation of non-fluorescent drug–protein complexes through static quenching mechanisms. This phenomenon is quantitatively characterized by the Stern–Volmer equation (Equation (3)) [29]:

(3) $$log(F_{0}-F)/F=nlogK_{a}-nlog[1/\left(\left[Q\right]-(F_{0}-F)\left[P\right]/F_{0}\right)]$$

in this context, *F*_0_ represents the fluorescence intensity of BSA when drugs are absent, while *F* signifies the intensity when drugs are present; the binding constant is denoted by *K*_a_, the quantity of binding sites is denoted by *n*, [*Q*] signifies the overall drug concentration, and [*P*] denotes the total BSA concentration.

The Δ*H*, Δ*S*, and Δ*G* values were determined through the van’t Hoff and Gibbs–Helmholtz equations, to identify the principal factor influencing the stability of the drug–BSA complex [30]:

(4) $$lnK_{a}=-∆H/RT+∆S/R$$

(5) ∆G=∆H−T∆S

in this context, *R* signifies the gas constant with a value of 8.314 J·mol^−1^ K^−1^, and *T* represents the absolute temperature (298, 303, and 310 K), respectively; Enthalpy change is denoted by Δ*H*, entropy change is represented by Δ*S*, and free energy change is signified by Δ*G*.

### 2.9. Molecular Docking Studies

To characterize the interactions between BSA and drug compounds, molecular docking was carried out using the crystal structure of BSA (PDB ID: 4F5S) retrieved from the RCSB Protein Data Bank (http://www.rcsb.org), with all solvent molecules removed. Binding site analysis was conducted using AutoDock Vina and AutoDock 4.2 (Scripps Research Institute, La Jolla, CA, USA), with the lowest-energy conformation selected as the optimal binding mode. Visualization and analysis of the docking results were performed using PyMOL 1.3r1 (DeLano Scientific, Palo Alto, CA, USA). This integrated computational approach provided detailed insights into the molecular interactions between BSA and the compounds under study.

### 2.10. Protocol for the Synthesis and Analysis of the Nanoparticles

#### 2.10.1. Synthesis of DOX@BSA, DOX–Cu@BSA, and DOX–BTZ@BSA Nanoparticles

DOX@BSA nanoparticles were fabricated using a controlled desolvation method [18]. Briefly, DOX (11.1 mg) was dissolved in deionized water (5 mL) and then titrated into a 2% BSA solution (pH 9.0) under continuous ethanol infusion (1 mL·min^−1^). After sequential addition of the DOX solution, the mixture was stirred for 30 min (Magnetic stirrer: FOUR E’S PowerHT, Guangzhou, China). Covalent crosslinking was subsequently initiated by adding 25% glutaraldehyde (16.8 µL), followed by aging for 8 h. The crude nanoparticles were collected by concentrating the solution to 1 mL and centrifuging at 14,000 rpm. The resulting pellet was redispersed in water and lyophilized (Freeze drier: BIOCOOL Pilot2-4M, Beijing, China). The redispersed nanoparticles were frozen at −20 °C for 8 h and subsequently lyophilized under a vacuum of 0.01 mBar with a programmed temperature control (−30 °C for 4 h, −20 °C for 4 h, −10 °C for 2 h, 0 °C for 2 h, 10 °C for 2 h, 20 °C for 2 h, 30 °C for 24 h). The resulting lyophilized powder was stored at −20 °C for further use.

The same protocol was applied to synthesize DOX–Cu@BSA and DOX–BTZ@BSA nanoparticles.

#### 2.10.2. Characterization of the Nanoparticles

Nanoparticle physicochemical characterization was conducted using standardized protocols. Dynamic light scattering (DLS) analysis for hydrodynamic diameter (intensity-weighted), polydispersity index (PDI), and surface charge measurements was performed with a Malvern Zetasizer Nano-1900 instrument (Malvern Panalytical, Westborough, MA, USA).

Morphological examination was achieved via transmission electron microscopy (TEM) using a Hitachi HT7800 microscope (Hitachi High-Tech Corporation, Tokyo, Japan).

Quantitative encapsulation efficiency (EE) and drug loading capacity (DL) were determined through UV–Vis spectrophotometry using validated calibration curves (500 nm for DOX–BTZ, 479 nm for DOX–Cu). Both DOX–Cu and DOX–BTZ are considered single drug entities. The calculations followed the equations: EE (%) = (mass of drug encapsulated/initial drug mass) × 100; DL (%) = (mass of drug encapsulated/total nanoparticle mass) × 100.

### 2.11. Drug Release Measurements

DOX–Cu@BSA or DOX–BTZ@BSA nanoparticles (2 mg) were uniformly dispersed in PBS containing 10% FBS at pH 7.4 or 5.7 at 4 °C. After centrifugation at 14,000 rpm, the pellets were resuspended in 2 mL of fresh PBS containing 10% FBS and subjected to continued agitation. Aliquots of the supernatant were collected at predetermined time intervals (0.5, 1, 2, 4, 6, 8, 10, 18, 24, and 48 h) and analyzed by UV–Vis spectroscopy (479 nm for DOX–BTZ, 500 nm for DOX–Cu) to determine the cumulative drug release profile [31]. Both DOX–Cu and DOX–BTZ are considered single drug entities.

### 2.12. Cellular Uptake

A549 human lung carcinoma cells were seeded onto glass-bottom confocal microscopy chambers. The culture plates were incubated in a humidified CO_2_ incubator at precisely 37 °C with 5.0% CO_2_ for adherence and logarithmic-phase proliferation. Following incubation, culture medium was carefully aspirated and replaced with fresh pre-warmed (37 °C) medium containing either DOX–Cu@BSA NPs or DOX–BTZ@BSA NPs. After 12 h incubation to enhance nanoparticle internalization, nuclei were labeled with DAPI. Uptake and distribution were observed via CLSM.

## 3. Results

### 3.1. Physicochemical Characterization

#### 3.1.1. The Study of DOX–Cu and DOX–BTZ Formation

**Figure 3:**
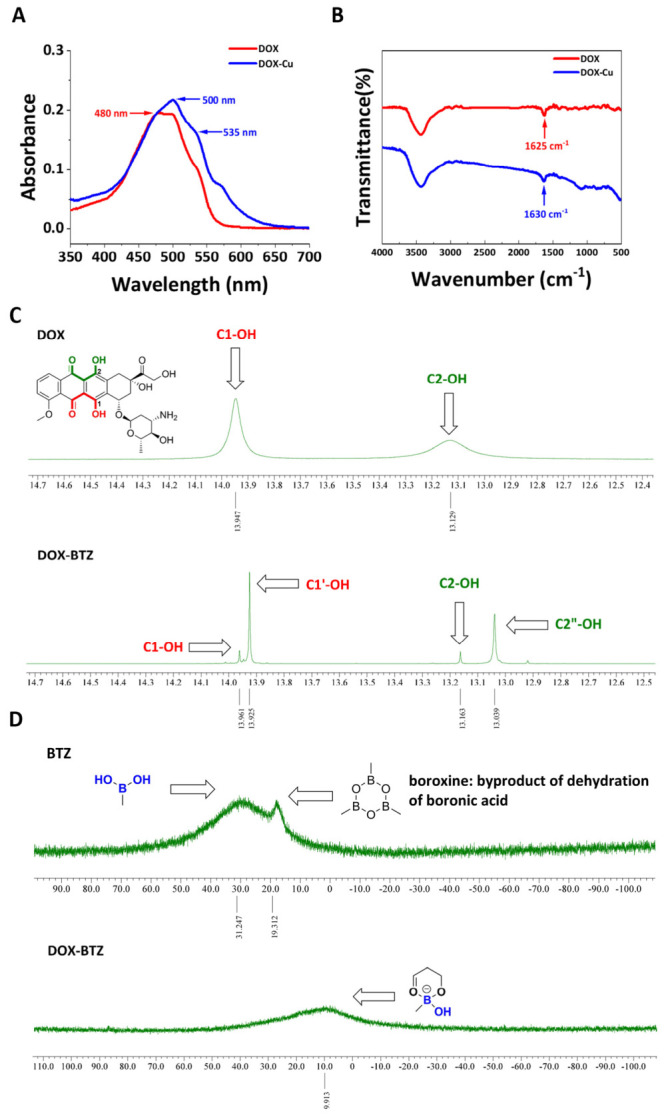
(**A**) UV–Vis absorption spectra of free DOX (red) and DOX–Cu (blue) at 25 °C, pH 7.4. (**B**) FT-IR spectra of free DOX (red) and DOX–Cu (blue). (**C**) ^1^H NMR spectra of DOX–BTZ in DMSO-*d*_6_ at 25 °C. (**D**) ^11^B NMR spectra of DOX–BTZ in DMSO-*d*_6_ at 25 °C.

The formation of the DOX–Cu complex was verified by UV–Vis and FT-IR spectroscopy [22,23]. UV–Vis analysis showed that the characteristic absorption of free DOX near 480 nm (Figure 3A) underwent a red shift upon addition of Cu^2+^, accompanied by the appearance of new absorption peaks, consistent with metal coordination [22]. FTIR spectra further indicated a shift in the C=O stretching vibration from 1625 cm^−1^ to 1630 cm^−1^ (Figure 3B), suggesting interaction between the carbonyl group and Cu^2+^ [23].

In the ^1^H NMR spectra, the hydroxyl proton signals of DOX at 13.95 ppm and 13.13 ppm decreased upon addition of BTZ, while new signals emerged at 13.93 ppm and 13.04 ppm (Figure 3C, Figure S1 and Figure S2) [32]. ^11^B NMR spectra of BTZ displayed a boronic acid signal at 31.25 ppm and a boroxine derivative signal at 19.31 ppm (Figure 3D) [33,34]. After complexation with DOX, the boron resonance shifted from 31.25 ppm to 9.91 ppm, indicating the formation of a tetra-coordinated boron structure (Figure 3D, Figure S3 and Figure S4) [35].

#### 3.1.2. Determination of Drug–BSA Interaction via Fluorescence Spectra

**Figure 4:**
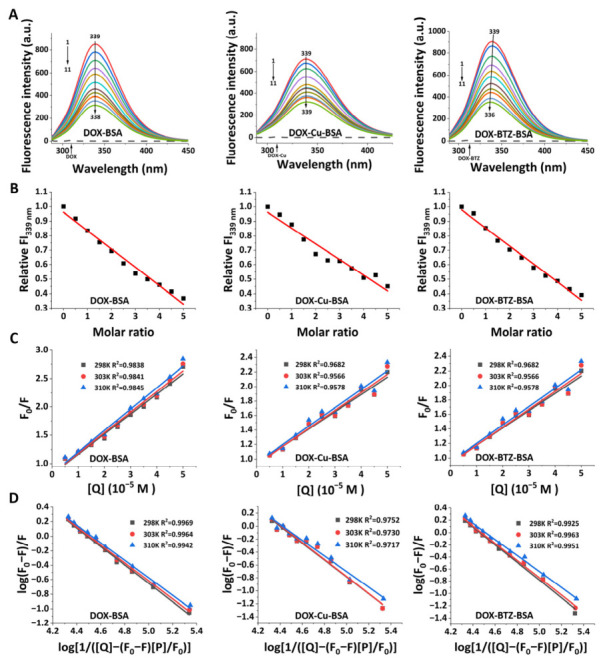
(**A**) Fluorescence emission spectra of BSA (5 μM) obtained at 25 °C/pH 7.4 with escalating drug concentrations. Spectra 1–11 correspond to drug levels of 0–50 μM (0, 5, 10, 15, 20, 25, 30, 35, 40, 45, 50). Bottom dotted line shows drug-only fluorescence. (**B**) The relative fluorescence intensity of BSA at 339 nm (Fl339 nm) as the molar ratio of the drugs to BSA rises. (**C**) Stern–Volmer curves for BSA fluorescence quenching by drug across varied temperatures. (**D**) Double-logarithmic plots of BSA fluorescence quenching by the drug at different temperatures.

The intrinsic fluorescence of bovine serum albumin (BSA), primarily from its trypot phan and tyrosine residues (*λ*_em_ ~339 nm) [28], was utilized to probe drug interactions. Concentration-dependent fluorescence quenching was observed upon the addition of drugs. Free drugs exhibited negligible intrinsic fluorescence (Figure 4A). At the highest DOX concentration (50 μM), BSA fluorescence intensity decreased by approximately 63%. Coordination with Cu(II) or formation of the BTZ–DOX complex resulted in further quenching of ~55% and ~58%, respectively, accompanied by spectral shifts (Figure 4B). Stern–Volmer analysis at 298, 303, and 310 K yielded linear plots (Figure 4C) with corresponding *K*_SV_ values (Table S1). The calculated bimolecular quenching rate constants (*k*_q_) ranged from 4.01 to 6.41 × 10^12^ L·mol^−1^·s^−1^ across these temperatures. Analysis of binding isotherms (Figure 4D) yielded binding constants (*K*_a_) on the order of ~10^4^ L·mol^−1^, which increased with temperature (Table S1). Thermodynamic parameters (Δ*H*, Δ*S*, Δ*G*) were derived from van’t Hoff plots (Table S1), revealing consistently negative Δ*G* values and positive values for both Δ*H* and Δ*S*.

#### 3.1.3. Three-Dimensional Fluorescence Spectra

**Figure 5:**
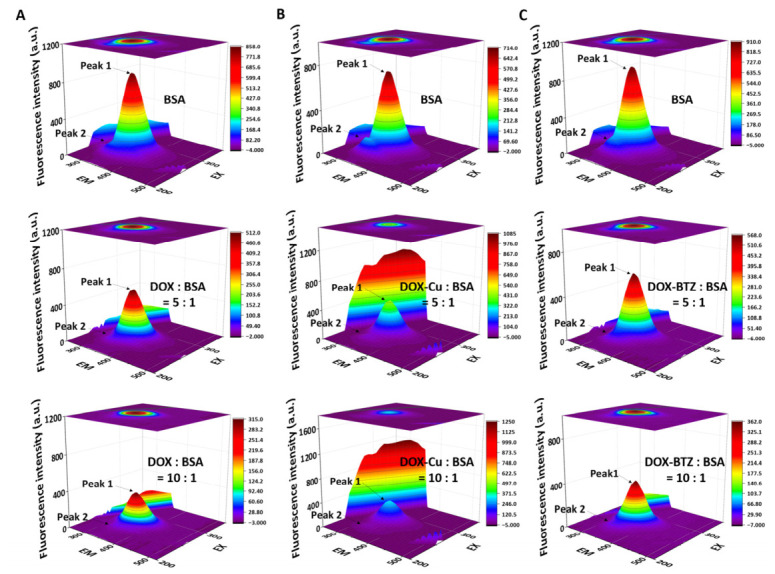
(**A**) 3D fluorescence spectral profiles of BSA (5 μM), DOX–BSA (5:1), and DOX–BSA (10:1) at 25 °C, pH 7.4. (**B**) 3D fluorescence spectral profiles of BSA (5 μM), (DOX–Cu)–BSA (5:1), and (DOX–Cu)–BSA (10:1) at 25 °C, pH 7.4. (**C**) 3D fluorescence spectral profiles of BSA (5 μM), (DOX–BTZ)–BSA (5:1), and (DOX–BTZ)–BSA (10:1) at 25 °C, pH 7.4.

Three-dimensional fluorescence spectral analysis of BSA revealed significant micro environmental alterations around its Tyr/Trp residues following drug interaction. The spectra and contour plots for BSA alone and in drug-complexed states are shown in Figure 5, with quantitative data detailed in Table S2. Peak 1, primarily associated with ligand binding at specific BSA sites, showed intensity reductions of 39% (DOX), 37% (DOX–Cu), and 36% (DOX–BTZ) at a 5:1 drug-to-BSA molar ratio. Under the same conditions, Peak 2, largely reflective of protein secondary structure and drug-induced conformational changes, exhibited greater quenching, with intensity decreases of 66% (DOX), 62% (DOX–Cu), and 60% (DOX–BTZ). These quenching effects were further intensified at a 10:1 molar ratio.

#### 3.1.4. Molecular Docking Studies

**Figure 6:**
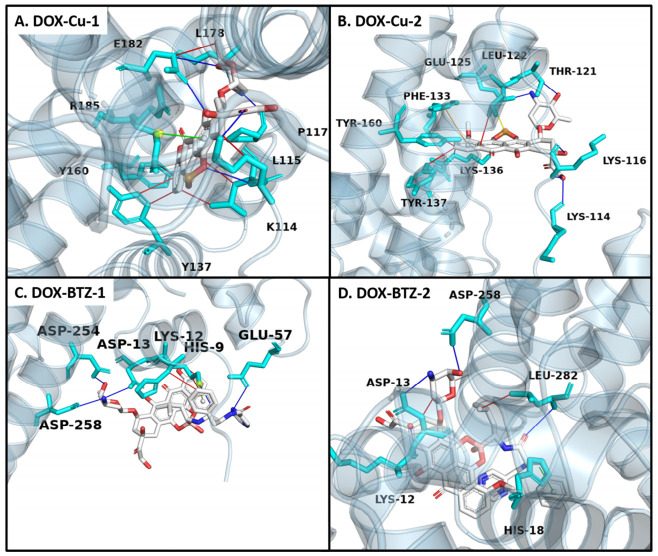
Docking analysis of drug–BSA complexes at distinct binding sites: DOX–Cu-1 (**A**) and DOX–Cu-2 (**B**) adopt lowest-energy conformations at site II (subdomain IIIA). Stick models illustrate key molecular interactions within the binding pocket, including hydrophobic contacts (red lines), hydrogen bonds (blue lines), π-cation interactions (green lines), π-stacking (orange) and metal coordination (yellow lines). DOX–BTZ-1 (**C**) and DOX–BTZ-2 (**D**) exhibit optimal binding at site III (subdomain IB). Detailed visualizations emphasize hydrophobic engagements (red), hydrogen linkages (blue) and π-cation interactions (green lines) reinforcing each complex.

Molecular docking simulations were performed to elucidate the binding modes of DOX, DOX–Cu, and DOX–BTZ complexes with bovine serum albumin (BSA) (Figure S6 and Figure 6). The ligands were optimized prior to docking into three predefined binding sites on BSA (site I/IIA, site II/IIIA, and site III/IB). The DOX–Cu complex exhibited a strong binding preference for site II (subdomain IIIA). Two representative conformations, DOX–Cu-1 and DOX–Cu-2, displayed high thermodynamic stability with calculated binding energies of −38.99 and −42.55 kJ·mol^−1^, respectively (Table S3, Figure 6A,B). The DOX–BTZ complex demonstrated site-specific binding, with DOX–BTZ-1 favoring site I and DOX–BTZ-2 favoring site II (Figure 6C,D), supported by binding energies of −28.16 and −30.42 kJ·mol^−1^, respectively (Table S3). Analysis of binding poses revealed that stabilization involved various non-covalent interactions, including hydrophobic contacts, hydrogen bonds, and π-cation/π-stacking interactions, with metal coordination also contributing to the DOX–Cu/DOX–BTZ complexes (Table S4).

#### 3.1.5. Characterization of the Nanoparticles

##### Physicochemical Characterization

**Figure 7:**
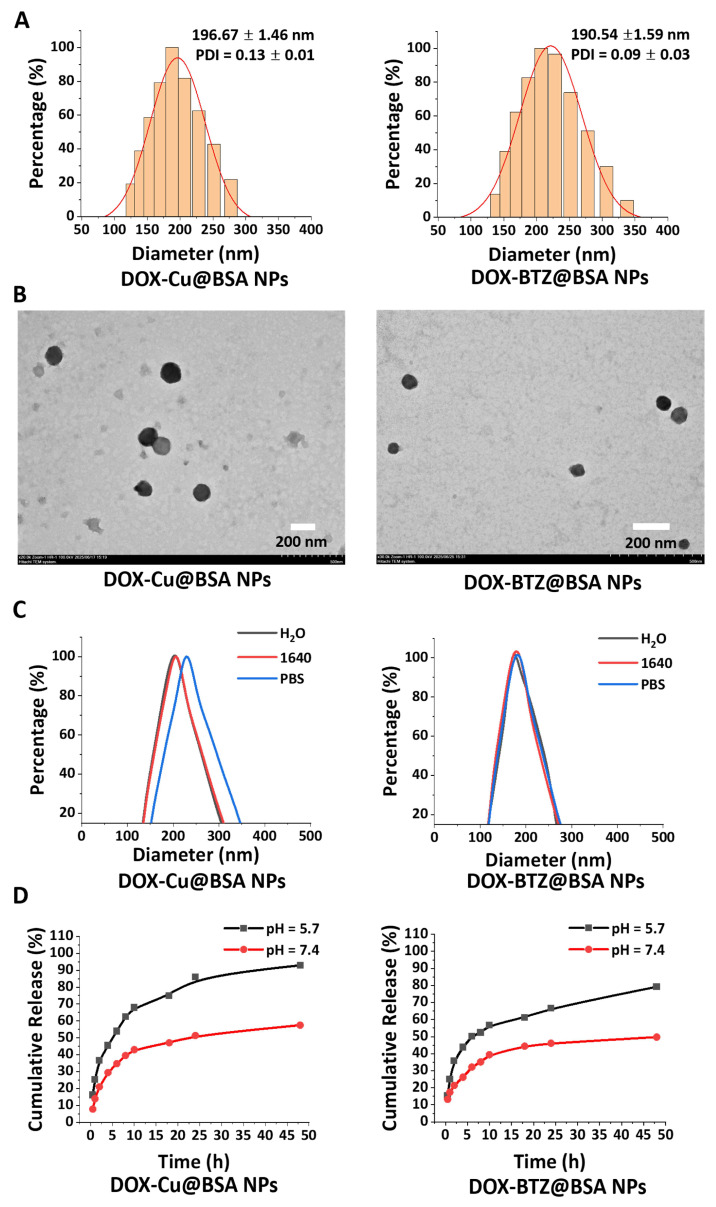
(**A**) Particle size distribution analysis for DOX–Cu@BSA NPs and DOX–BTZ@BSA NPs (*n* = 3, independent batches). (**B**) TEM images of DOX–Cu@BSA NPs and DOX–BTZ@BSA NPs. (**C**) Structural stability assessment of DOX–Cu@BSA NPs and DOX–BTZ@BSA NPs (*n* = 3, independent measurements). (**D**) Cumulative release dynamics in PBS containing 10% FBS (*n* = 3, independent measurements).

DOX–Cu@BSA and DOX–BTZ@BSA nanoparticles (NPs) were synthesized using an optimized systematic desolvation technique, with key parameters including water/ethanol ratio, infusion rate, pH, BSA concentration, and crosslinking duration fine-tuned (Tables S5–S14). The resulting NPs exhibited uniform spherical morphology with an average diameter of ~190 nm, a narrow size distribution (Figure 7A, Table 1), and a zeta potential of approximately −10 mV (Table 1).

##### Drug Loading and Encapsulation Efficiency

Their spherical shape and monodispersity were confirmed by TEM (Figure 7B). Encapsulation efficiency (EE) and drug loading (DL) were 74.55%/7.74% for DOX–Cu@BSA NPs and 78.77%/7.86% for DOX–BTZ@BSA NPs, respectively (Table 1). Both NP formulations maintained their particle size without significant change over 24 h in deionized water, PBS (pH 7.4), or RPMI 1640 medium (Figure 7C, Table 2). The slight increase in PDI for DOX–Cu@BSA NPs in PBS (pH 7.4) after 24 h may be attributed to mild aggregation under physiological ionic strength, whereas NPs remained stable in deionized water and culture medium.

##### In Vitro Drug Release Profiles

In vitro drug release studies revealed distinct profiles: at physiological pH (7.4), DOX–Cu@BSA NPs showed a sustained, diffusion-controlled release (57% cumulative release at 48 h), while DOX–BTZ@BSA NPs released 49% over the same period. Under acidic conditions (pH 5.7), both NPs exhibited accelerated release within 48 h, with DOX–Cu@BSA NPs of 92% and DOX–BTZ@BSA NPs of 79%. Comparable pH-dependent release trends were observed in plasma-fortified media, though with slightly attenuated kinetics, underscoring the robustness of the release mechanism under biorelevant conditions (Figure 7D).

### 3.2. Biological Evaluation

#### 3.2.1. Effects of the DOX–Cu and DOX–BTZ Complexes on A549 Cells

**Figure 8:**
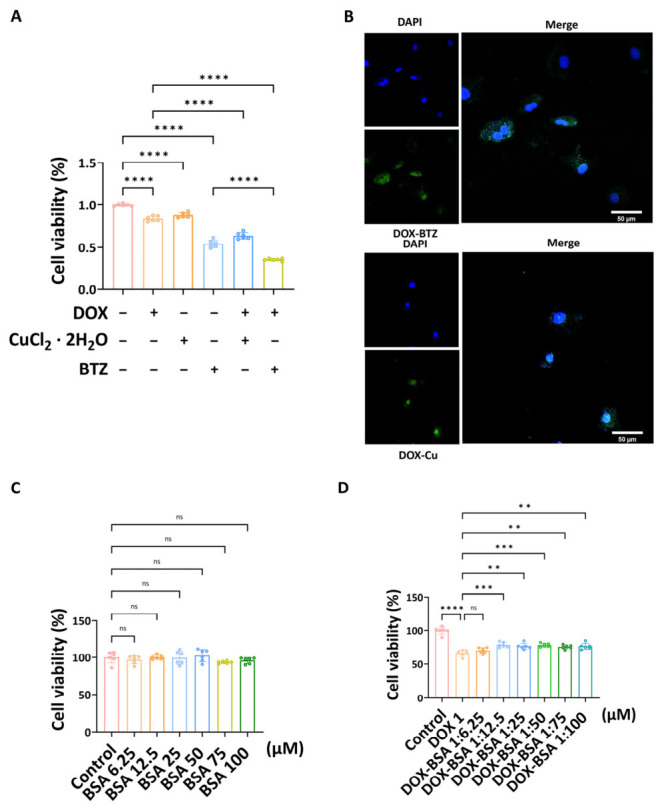
(**A**) CCK-8 assay measuring A549 cell viability after treatment with various agents at 0.0625 µM (*n* = 6, independent experiments). (**B**) Confocal images showing DLAT aggregation in A549 cells treated with DOX complexes. Acquired with a 40× oil immersion objective. Scale bar = 50 µm (*n* = 3, independent experiments). (**C**) Viability of AC16 cardiomyocytes treated with BSA alone (6.25–100 µM) (*n* = 6, independent experiments). (**D**) Viability of AC16 cells treated with DOX–BSA complexes at fixed DOX concentration (1 µM) and varying BSA concentrations (*n* = 6, independent experiments). ns denotes not significant (*p* ≥ 0.05), ** denotes *p* < 0.01, *** denotes *p* < 0.001, and **** denotes *p* < 0.0001.

CCK-8 assays demonstrated that the DOX–BTZ complex significantly reduced the viability of A549 cells (Figure 8A). The combined treatment of DOX and BTZ exhibited stronger cytotoxic effects compared to DOX alone. Furthermore, the DOX–Cu chelate, formed by combining DOX with CuCl_2_·2H_2_O, also showed an enhanced inhibitory effect on A549 cell viability.

#### 3.2.2. DOX–Cu and DOX–BTZ Complexes Triggers Cell Death Associated with Cuproptosis in A549 Cells

Immunofluorescence analysis was performed to assess DLAT aggregation in A549 cells. As shown in Figure 8B and Figure S5, untreated cells and those treated with DOX alone displayed only faint green fluorescence. In contrast, cells treated with either the DOX–BTZ or DOX–Cu complex exhibited a marked intensification of fluorescence signal, indicating substantial DLAT aggregation.

#### 3.2.3. Protective Effects of the BSA Complex on AC16

Cardiomyocytes (AC16 cells) were treated with varying concentrations of BSA (6.25 to 100 μM) in the absence of DOX. CCK-8 assays indicated that BSA alone, at all tested concentrations, had no significant impact on cardiomyocyte viability compared to the control (Figure 8C). The protective effect of the DOX–BSA complex against DOX-induced toxicity was subsequently evaluated. While co-treatment with 1 μM DOX and 6.25 μM BSA showed no significant difference in cell viability compared to DOX alone, complexes containing higher BSA concentrations (12.5 to 100 μM) significantly enhanced AC16 cell viability (Figure 8D). Overall, cells treated with the DOX–BSA complex showed significantly higher viability compared to those treated with free DOX, indicating a cardioprotective effect. We recognize that the 24 h treatment of AC16 cells represents an acute exposure model. Chronic or repeated-dose studies are needed to fully evaluate the long-term cardioprotective efficacy of these BSA nanoparticles, and such investigations are currently underway in our laboratory.

#### 3.2.4. Cellular Uptake, DLAT Aggregation and Cell Viability

**Figure 9:**
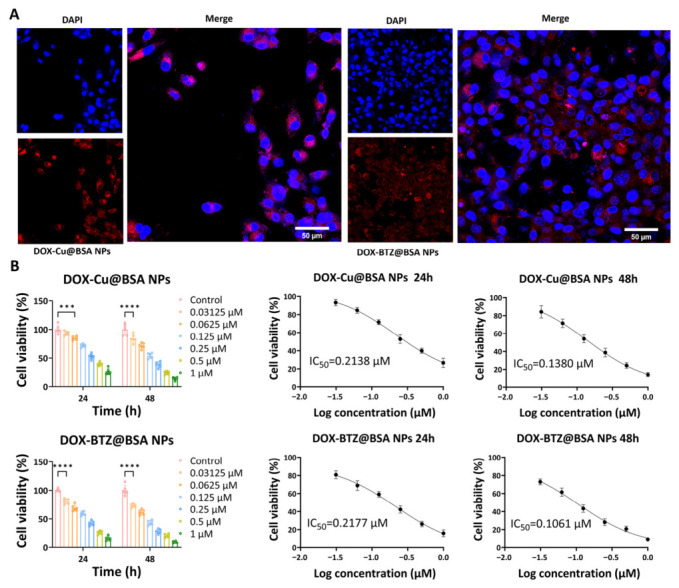
(**A**) CLSM images of A549 cells after 12 h incubation with DOX–Cu@BSA NPs or DOX–BTZ@BSA NPs. Red fluorescence indicates DOX from the internalized nanoparticles. Blue fluorescence (DAPI) stains cell nuclei. Acquired with a 40× objective. Scale bar = 50 µm (*n* = 3, independent experiments). (**B**) CCK-8 analysis measured cell viability of nanoparticles following varied dosage treatments (*n* = 6, independent experiments). *** denotes *p* < 0.001, **** denotes *p* < 0.0001.

Cellular internalization in A549 cells, assessed via fluorescence microscopy after 12 h co-incubation, showed clear perinuclear red fluorescence signals (Figure 9A), confirming efficient cellular uptake of both NP types. The CCK-8 assay demonstrated that both DOX–Cu@BSA NP and DOX–BTZ@BSA NP treatments reduced cell viability in a dose- and time-dependent manner (Figure 9B). Increasing concentrations from 0.03125 µM to 1 µM resulted in progressively lower viability at both 24 h and 48 h, with a more pronounced cytotoxic effect observed at the later time point. The calculated IC_50_ values further quantified this effect: for DOX–Cu@BSA NPs, the IC_50_ decreased from 0.2138 µM at 24 h to 0.1380 µM at 48 h; for DOX–BTZ@BSA NPs, it decreased from 0.2177 µM at 24 h to 0.1061 µM at 48 h. At 24 h, the two treatments exhibited nearly identical potency, whereas at 48 h DOX–BTZ@BSA NPs displayed a slightly lower IC_50_ than DOX–Cu@BSA NPs, suggesting enhanced cytotoxicity upon prolonged exposure.

**Figure 10:**
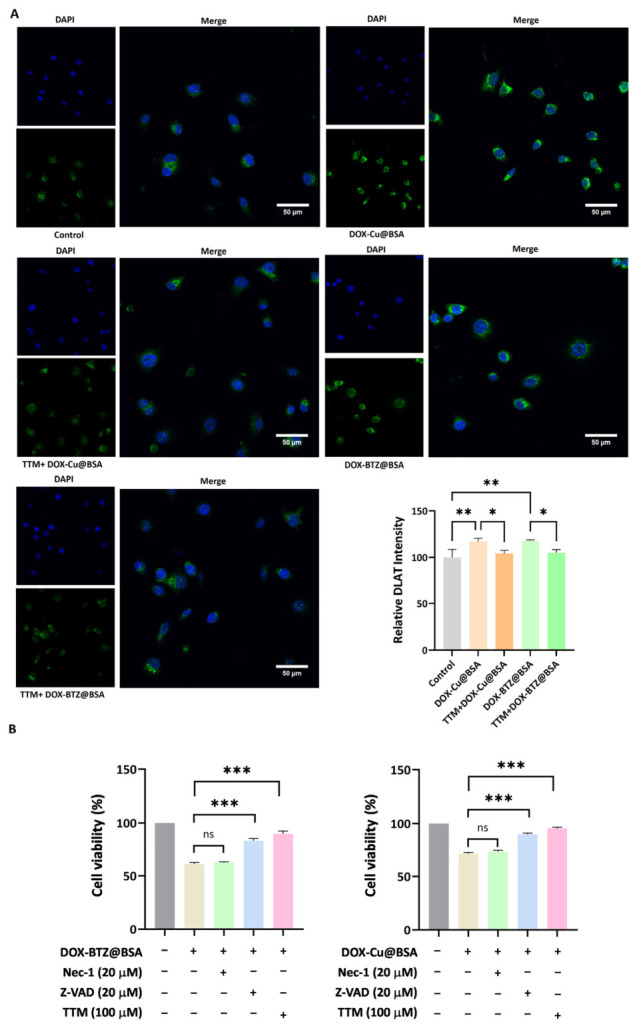
(**A**) CLSM images reflect DLAT aggregation in A549 cells after treated with the nanoparticles. Acquired with a 40× oil immersion objective. Scale bar = 50 µm (*n* = 3, independent experiments). (**B**) Viability of A549 cells after different treatments, DOX–BTZ@BSA: 0.0625 µM, DOX–Bu@BSA: 0.0625 µM, Nec-1: 20 µM, Z-VAD: 20 µM, TTM: 100 µM (*n* = 3, independent experiments). ns denotes not significant (*p* ≥ 0.05), * denotes *p* < 0.05, ** denotes *p* < 0.01, and *** denotes *p* < 0.001.

Immunofluorescence analysis of DLAT accumulation revealed that all treatment groups significantly increased relative DLAT intensity compared to the control group (Figure 10A). Among these, the DOX–Cu@BSA NPs group exhibited the highest level of DLAT accumulation, with a highly significant difference. The TTM+DOX–Cu@BSA NPs, DOX–BTZ@BSA NPs, and TTM+DOX–BTZ@BSA NPs groups also showed significant increases relative to the control. Notably, the addition of TTM attenuated the DLAT accumulation induced by DOX–Cu@BSA NPs, as reflected by the reduced significance level, whereas TTM did not markedly alter the effect of DOX–BTZ@BSA NPs. The CCK-8 assay was performed to evaluate the viability of cells treated with DOX–BTZ@BSA NPs or DOX–Cu@BSA NPs in the presence of various cell death inhibitors (Figure 10B). For both nanoparticles, co-treatment with the necroptosis inhibitor Nec1 did not significantly affect cell viability, whereas the pan-caspase inhibitor Z-VAD and the copper chelator TTM both led to a marked increase in cell viability.

## 4. Discussion

The red shift and emergence of new absorption peaks in the UV–Vis spectra confirm the coordination between DOX and Cu^2+^, reflecting a decrease in electronic transition energy upon complexation [22]. The shift in the C=O stretching frequency in the FT-IR spectrum further supports the involvement of the carbonyl group in chelation [23]. The downfield shift in the ^11^B NMR from 31.25 ppm to 9.91 ppm is consistent with the formation of a tetra-coordinated boron center, confirming the successful conjugation of DOX and BTZ [33,34,35].

The enhanced cytotoxicity of DOX–BTZ and DOX–Cu complexes in A549 cells, relative to their individual components, suggests a cooperative effect [36]. The DOX–BTZ conjugate may promote intracellular copper accumulation by stabilizing hCTR1, while the DOX–Cu complex directly supplies both copper and a chemotherapeutic agent. The marked aggregation of DLAT—a key marker of cuproptosis—further supports the involvement of this copper-dependent cell death pathway in the observed antitumor activity [37].

In cardiomyocytes, BSA complexation significantly attenuated DOX-induced cytotoxicity in a concentration-dependent manner, consistent with the role of albumin in modulating drug distribution and reducing off-target toxicity [38,39,40,41]. The fluorescence quenching and thermodynamic analyses revealed spontaneous binding between the complexes and BSA, with positive Δ*H* and Δ*S* values indicating that hydrophobic interactions are the primary driving force. These molecular interactions provide a mechanistic basis for the stability and sustained release properties of the nanoparticle formulations [42,43,44,45,46,47,48,49,50,51].

The DOX–Cu@BSA and DOX–BTZ@BSA nanoparticles exhibited uniform size (~190 nm), negative surface charge, and high encapsulation efficiency. Their pH-responsive release behavior—accelerated under acidic conditions (pH 5.7)—is particularly advantageous for tumor-targeted delivery, leveraging the acidic tumor microenvironment to trigger localized drug release [52]. Efficient cellular internalization of both nanoparticle types was confirmed in A549 cells.

The time- and dose-dependent reduction in cell viability, along with the decrease in IC_50_ from 24 to 48 h, indicates sustained cytotoxic activity. Immunofluorescence analysis revealed that both nanoparticle formulations induced DLAT aggregation, which was attenuated by the copper chelator TTM, further implicating cuproptosis in the mechanism of action. The partial rescue of cell viability by the pan-caspase inhibitor Z-VAD suggests that apoptosis also contributes to the overall cell death, consistent with the known pro-apoptotic effects of DOX. In contrast, the necroptosis inhibitor Nec-1 had no significant effect, indicating that necroptosis is not a major pathway in this context.

While the current findings are derived from in vitro experiments, they provide a strong proof-of-concept for the use of BSA-based nanocarriers to deliver doxorubicin-based complexes that induce cuproptosis. Ongoing in vivo studies will be essential to evaluate the pharmacokinetics, tumor-targeting efficiency, and therapeutic safety of these formulations.

## 5. Conclusions

In summary, we demonstrated that doxorubicin-based complexes, specifically DOX–Cu and DOX–BTZ, effectively induce cuproptosis in A549 lung cancer cells. Spectroscopic and NMR analyses confirmed the formation of these complexes, with DOX–Cu facilitating copper-mediated cytotoxicity and DOX–BTZ enhancing proteasome inhibition and copper accumulation. Both complexes promoted significant DLAT aggregation, a hallmark of cuproptosis, highlighting their potential as novel anticancer agents. Furthermore, BSA was employed to construct nano-delivery systems (DOX–Cu@BSA and DOX–BTZ@BSA NPs), which exhibited favorable physicochemical properties, efficient cellular uptake, and clear pH-responsive drug release in vitro, including in plasma-supplemented environments. Importantly, BSA complexation reduced the cardiotoxicity of doxorubicin in AC16 cardiomyocytes, underscoring its protective role. Molecular docking and fluorescence quenching studies revealed strong and spontaneous binding between the complexes and BSA, driven primarily by hydrophobic interactions. These findings collectively support the development of albumin-based multidrug delivery systems as a promising strategy for enhancing therapeutic efficacy and reducing systemic toxicity in lung cancer treatment. Future studies should focus on in vivo pharmacokinetic and efficacy evaluations to validate the therapeutic potential and pH-responsive behavior of these NPs in biological systems.

## Figures and Tables

**Table 1:** Particle size, zeta potential, EE and DL (*n* = 3, independent batches).

NPs	Particle Size (nm)	Zeta Potential (mV)	EE (%)	DL (%)
DOX–Cu@BSA	196.67 ± 1.46	−10.49 ± 0.89	74.55	7.74
DOX–BTZ@BSA	190.54 ± 1.59	−10.10 ± 1.94	78.77	7.86

**Table 2:** Stability of DOX–Cu@BSA NPs and DOX–BTZ@BSA NPs (*n* = 3, independent measurements).

NPs	System	Particle Size (0 h, nm)	PDI	Particle Size (24 h, nm)	PDI
	ddH_2_O	203.14 ± 2.03	0.04 ± 0.03	205.12 ± 1.58	0.08 ± 0.002
DOX–Cu@BSA	PBS (pH 7.4)	228.17 ± 2.90	0.06 ± 0.005	218.23 ± 3.64	0.17 ± 0.02
	RPMI 1640	209.61 ± 1.31	0.09 ± 0.01	208.63 ± 1.54	0.15 ± 0.03
	ddH_2_O	191.15 ± 0.47	0.11 ± 0.01	189.20 ± 0.72	0.14 ± 0.02
DOX–BTZ@BSA	PBS (pH 7.4)	165.57 ± 6.05	0.14 ± 0.06	177.07 ± 3.64	0.12 ± 0.01
	RPMI 1640	149.50 ± 1.00	0.25 ± 0.02	150.13 ± 1.60	0.25 ± 0.01

## Data Availability

The original contributions presented in this study are included in the article and Supplementary Material. Further inquiries can be directed to the corresponding authors.

## Supplementary Materials

The following supporting information can be downloaded at: https://www.mdpi.com/article/10.3390/pharmaceutics18050526/s1, Figure S1: ^1^H NMR spectrum of DOX in DMSO-*d*_6_; Figure S2: ^1^H NMR spectrum of BTZ-DOX in DMSO-*d*_6_; Figure S3: ^11^B NMR spectrum of BTZ in DMSO-*d*_6_; Figure S4: ^11^B NMR spectrum of BTZ-DOX in DMSO-*d*_6_; Figure S5: Immunofluorescence staining images reflecting DLAT aggregation in the A549 cells after the indicated treatments; Figure S6: Binding orientations of the lowest docking energy conformation of DOX (rendered in sticks) between binding site III (subdomain IB) of BSA (Left). The enlarged view of the binding locus showing the hydrophobic interaction (red line) and hydrogen bonds (blue line) (Right); Figure S7: Cumulative release dynamics in PBS across varied conditions (*n* = 3, independent measurements). (A) DOX-Cu@BSA NPs; (B) DOX-BTZ@BSA NPs; Figure S8: CCK-8 analysis measured cell viability following varied treatments (*n* = 6, independent experiments). The dosing concentration was fixed at 0.0625 µM. **** denotes *p* < 0.0001.; Table S1: The quenching constant (*K*_sv_), biomolecular quenching rate constant (*k*_q_), binding constants (*K*_a_) and thermodynamic parameters of BSA by the drugs at three different temperatures, pH 7.4; Table S2: 3D Fluorescence spectral data of BSA (5 μM) and the drugs at 25 °C, pH 7.4; Table S3: Docking results of Drug-BSA complex; Table S4: Key molecular interactions within the binding pocket; Table S5: Effect of Water-to-Ethanol Ratio on the formation of DOX-Cu@BSA NPs; Table S6: Effect of drop rate of ethanol on the formation of DOX-Cu@BSA NPs; Table S7: Effect of pH value on the formation of DOX-Cu@BSA NPs; Table S8: Effect of BSA concentration on the formation of DOX-Cu@BSA NPs; Table S9: Effect of crosslinking time on the formation of DOX-Cu@BSA NPs; Table S10: Effect of Water-to-Ethanol Ratio on the formation of DOX-BTZ@BSA NPs; Table S11: Effect of drop rate of ethanol on the formation of DOX-BTZ@BSA NPs; Table S12: Effect of pH value on the formation of DOX-BTZ@BSA NPs; Table S13: Effect of BSA concentration on the formation of DOX-BTZ@BSA NPs; Table S14: Effect of crosslinking time on the formation of DOX-BTZ@BSA NPs.

## Abbreviations

The following abbreviations are used in this manuscript:

DOX	Doxorubicin
BTZ	Bortezomib
BSA	Bovine serum albumin
HSA	Human serum albumin
DLAT	Dihydrolipoamide *S*-acetyltransferase
hCTR1	Human copper transporter 1
NMR	Nuclear magnetic resonance spectrum
UV–Vis	Ultraviolet–visible absorption spectrum
FT-IR	Fourier transform infrared spectroscopy
TEM	Transmission electron microscopy
CLSM	Confocal laser scanning microscopy
CCK-8	Cell Counting Kit-8
Z-VAD	Z-VAD(OH)-FMK
Nec-1	Necrostatin-1
TTM	Ammonium tetrathiomolybdate
