# Supplementary material for: pH-Responsive Bovine Serum Albumin Nanoparticles Encapsulating Doxorubicin-Based Complexes Induce Cuproptosis in Lung Cancer Cells
Source: Pharmaceutics. 2026 Apr 26;18(5):526. doi: 10.3390/pharmaceutics18050526 (PMC13210156; doi:10.3390/pharmaceutics18050526)
Supplement: Supplementary file 1 [file pharmaceutics-18-00526-s001.zip › pharmaceutics-4181438-supplementary.pdf]

Supplementary Material

pH-Responsive Bovine Serum Albumin Nanoparticles Encapsulating Doxorubicin-Based Complexes Induce Cuproptosis in Lung Cancer Cells

Haiying Zhang,[1,2,#] Xuanjia Chen,[1,#] Shihui Qiao,[1] Huanfeng Meng,[1] Hui Long,[1,2] Huamin Zhong,[1] Yiheng Liu,[3] Yun Song,[1] Yanan Gao,[1] Yan Liu,[1,*] Lujia Mao[1,*]

[1] Hainan Provincial Key Laboratory of Research and Development on Tropical Herbs, Engineering Research Center of Tropical Medicine Innovation and Transformation of Ministry of Education, Key Laboratory of Tropical Translational Medicine of Ministry of Education, Haikou Key Laboratory of Li Nationality Medicine, College of Pharmacy/College of Basic Medical Sciences, Hainan Academy of Medical Science, Hainan Medical University, Haikou 571199, Hainan, P. R. China

[2] Biobank, Hainan Medical University, Haikou 571199, Hainan, P. R. China

[3] Affiliated Haikou Hospital of Xiangya Medical College, Central South University, Haikou 570208, Hainan, P. R. China

E-mail: hy0207117@muhn.edu.cn (Y. L.); maolujia@muhn.edu.cn (L. M.).

S1. NMR Spectra

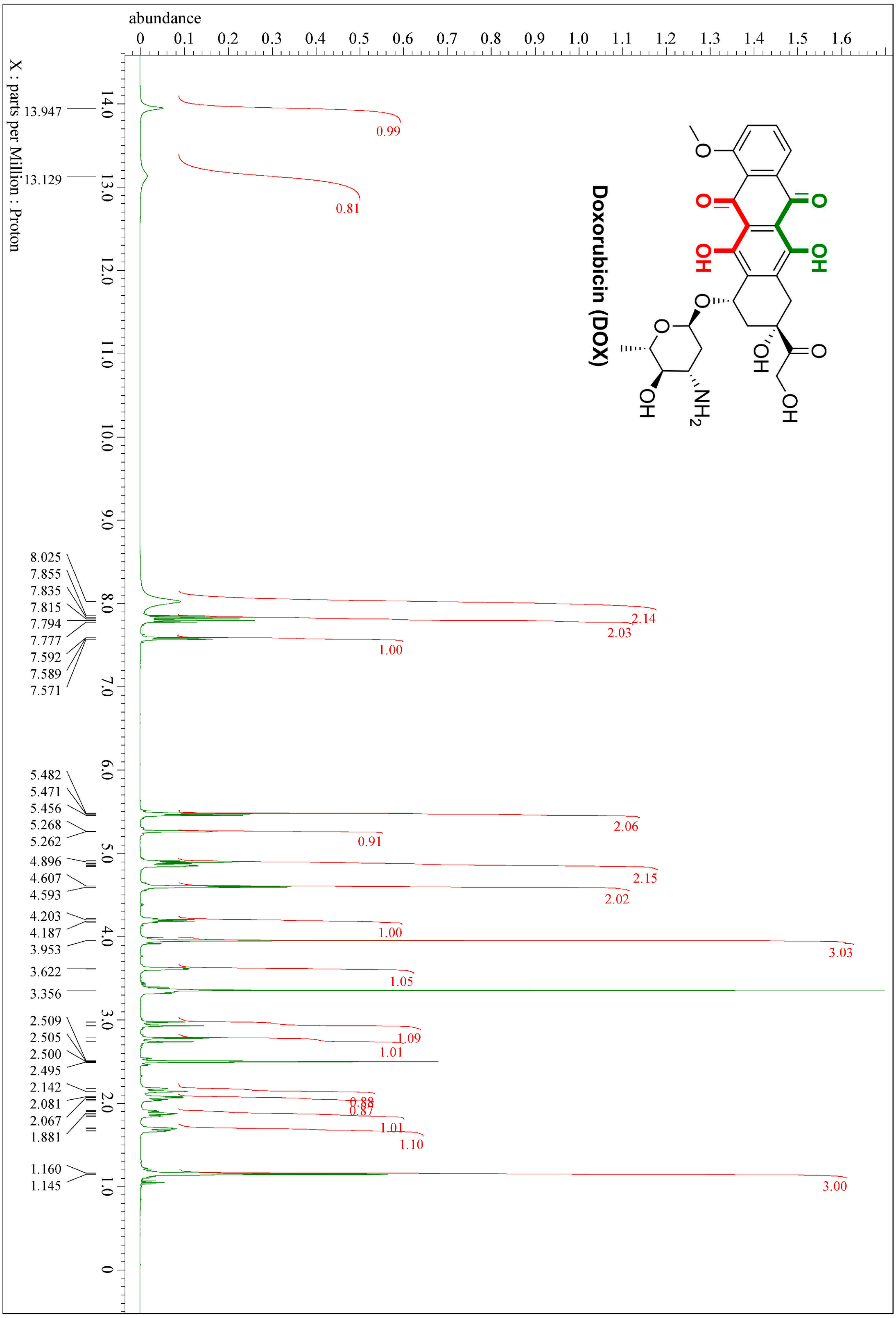

Figure S1. ^1H NMR spectrum of DOX in DMSO-d_6.

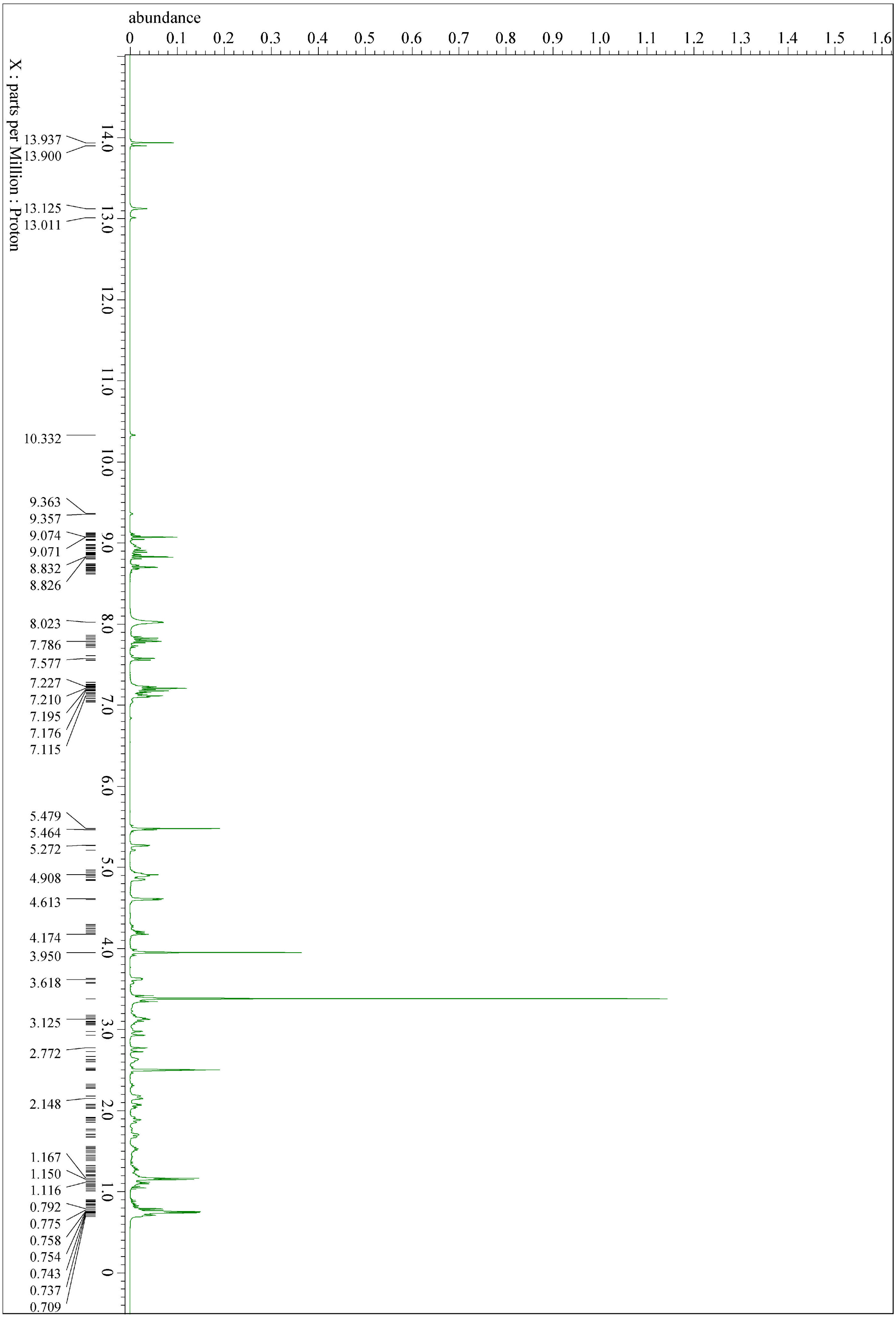

Figure S2. ^{1}H NMR spectrum of BTZ-DOX in DMSO-d_6.

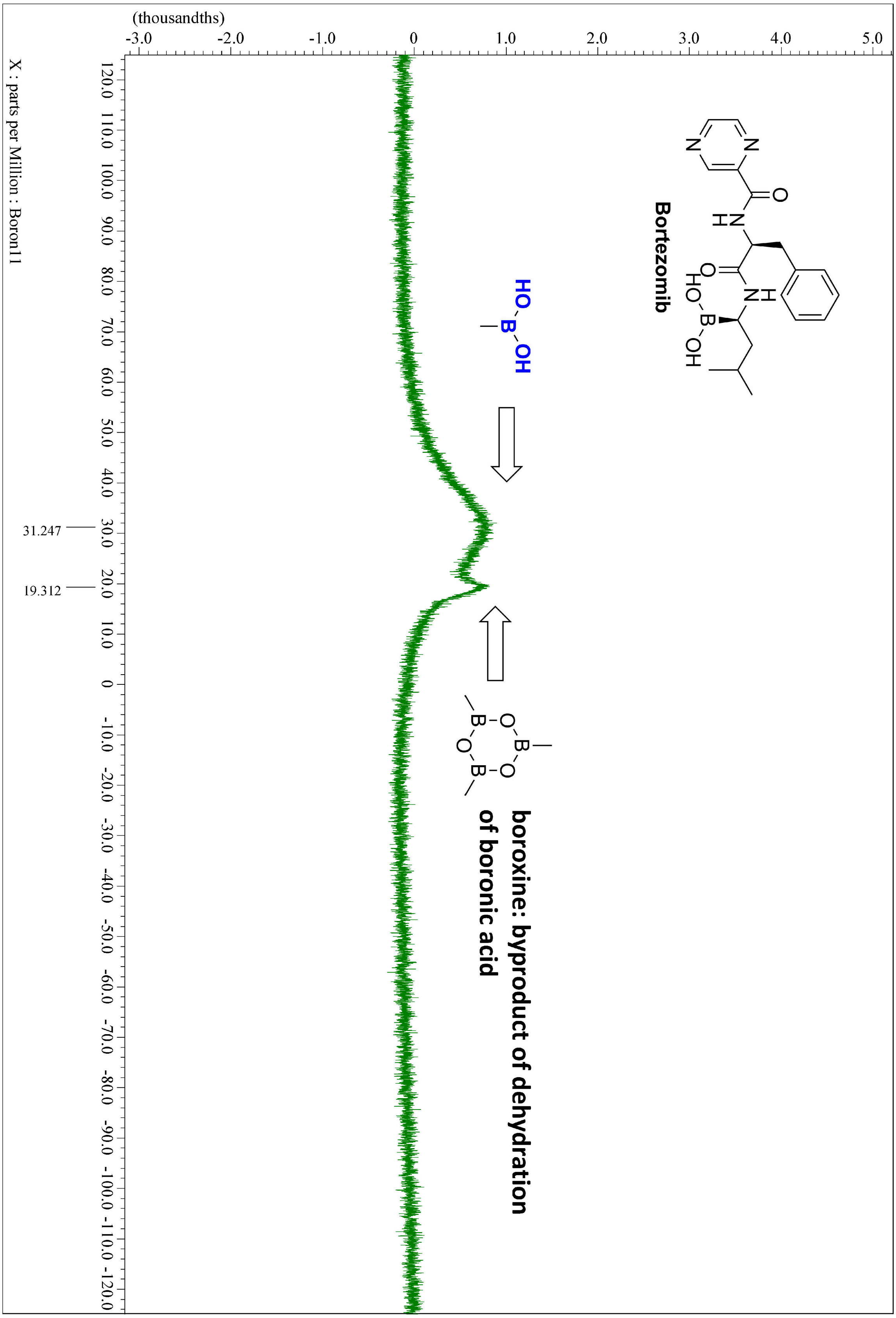

Figure S3. ^{11}B NMR spectrum of BTZ in DMSO-d_6.

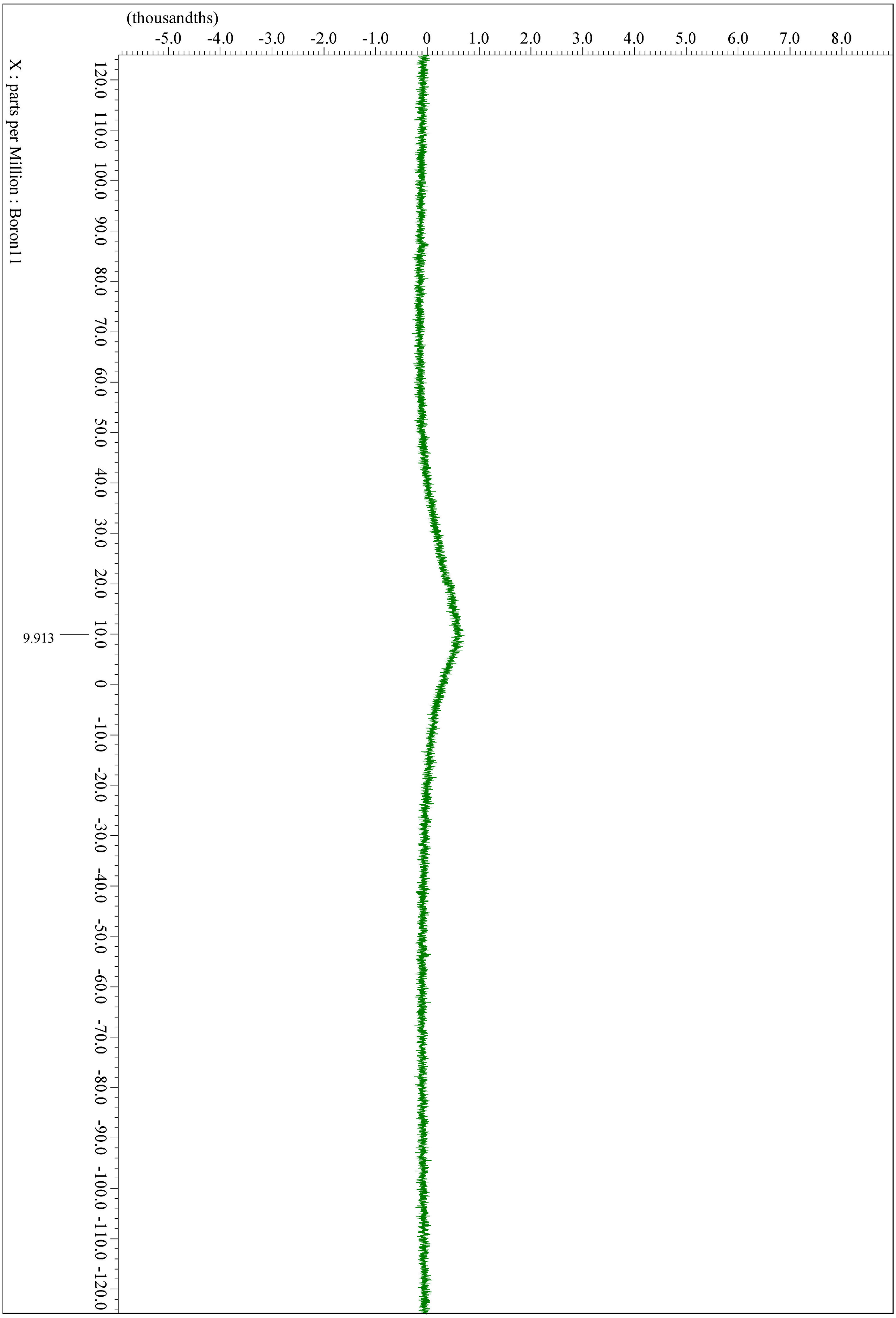

Figure S4. ^{11}B NMR spectrum of BTZ-DOX in DMSO-d_6.

S2. IF analysis of DLAT aggregation in A549 cells

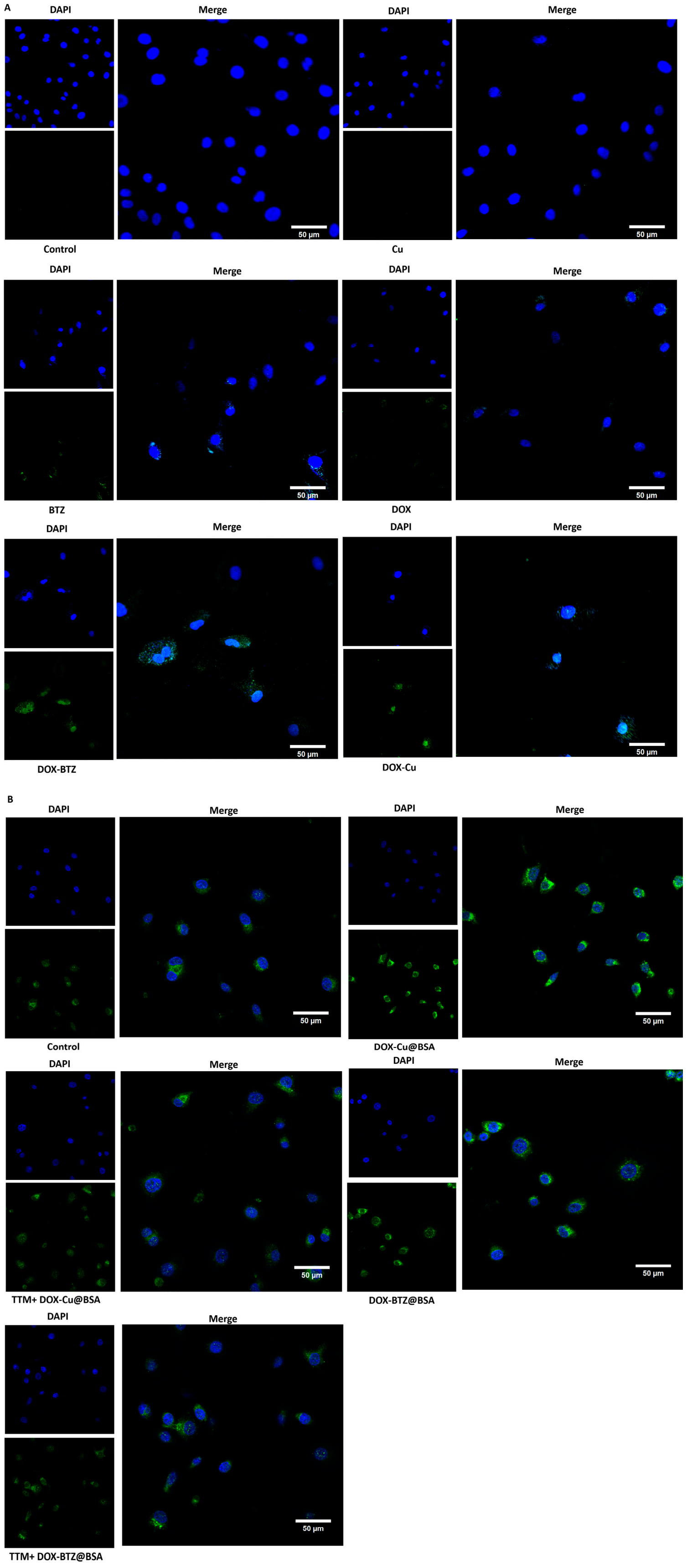

Figure S5. Immunofluorescence staining images reflecting DLAT aggregation in the A549 cells after the indicated treatments.

S3. Drug-BSA Interaction

S3.1. Fluorescence spectra and quenching mechanism

Table S1. The quenching constant (K_{sv}), biomolecular quenching rate constant (k_q), binding constants (K_a) and thermodynamic parameters of BSA by the drugs at three different temperatures, pH 7.4.

Drug	T (K)	K_{sv} ×10^4 (L·mol^{-1})	k_q ×10^{12} (L·mol^{-1} s^{-1})	K_a ×10^4 (L·mol^{-1})	n	ΔS (J·mol^{-1}·K^{-1})	ΔH (kJ·mol^{-1})	ΔG (kJ·mol^{-1})
	298	3.56±0.033	5.94±0.055	3.10±0.013	1.28±0.004			-36.95
DOX	303	3.63±0.002	6.06±0.003	3.19±0.004	1.25±0.012	+150.04	+7.77	-37.70
	310	3.82±0.034	6.36±0.064	3.38±0.017	1.22±0.013			-38.75
	298	2.41±0.016	4.01±0.026	2.52±0.004	1.29±0.021			-36.21
DOX-Cu	303	2.42±0.057	4.03±0.095	2.55±0.022	1.37±0.044	+136.02	+4.33	-36.89
	310	2.55±0.007	4.25±0.011	2.64±0.002	1.23±0.016			-37.84
	298	3.28±0.007	5.46±0.011	2.90±0.004	1.45±0.016			-36.70
DOX-BTZ	303	3.47±0.020	5.78±0.033	2.99±0.005	1.41±0.017	+160.26	+11.05	-37.50
	310	3.84±0.012	6.41±0.020	3.27±0.005	1.31±0.008			-38.62

S3.2. Three-dimensional fluorescence spectra

Table S2. 3-D Fluorescence spectral data of BSA (5 mol·L^{-1}) and the drugs at 25 ^{o}C, pH 7.4.

System	Peak	Peak position [λ_{ex}/λ_{em} (nm/nm)]	Intensity
BSA	1	280/339	838.62
	2	240/340	173.09
[DOX] : [BSA] = 5:1	1	280/339	509.30
	2	240/340	58.91
[DOX] : [BSA] = 10:1	1	280/338	312.38
	2	240/338	22.16
BSA	1	280/339	699.88
	2	235/340	165.88
[DOX-Cu] : [BSA] = 5:1	1	280/339	441.71
	2	235/340	63.04
[DOX-Cu] : [BSA] = 10:1	1	280/339	316.35
	2	235/335	50.35
BSA	1	280/339	884.47
	2	240/340	206.47
[DOX-BTZ] : [BSA] = 5:1	1	280/339	567.36
	2	240/340	82.04
[DOX-BTZ] : [BSA] = 10:1	1	280/335	344.00
	2	240/340	32.39

S3.3. Molecular docking studies

Table S3. Docking results of Drug-BSA complex.

System	Binding site	ΔG^0 (kJ·mol^{-1})^a	ΔE_1 (kJ·mol^{-1})^b	ΔE_2 (kJ·mol^{-1})^c	ΔE_3 (kJ·mol^{-1})^d
	Site I	-30.12	-41.59	-39.16	-2.43
**DOX-Cu-1**	Site II	-38.99	-50.50	-47.15	-3.31
	Site III	-37.61	-49.12	-30.38	-18.70
	Site I	-39.75	-51.21	-41.55	-9.67
**DOX-Cu-2**	Site II	-42.55	-54.06	-43.89	-10.13
	Site III	-34.60	-46.11	-35.56	-10.50
	Site I	-18.32	-41.25	-38.15	-3.10
**DOX-BTZ-1**	Site II	-22.68	-45.65	-40.17	-5.52
	Site III	-28.16	-51.13	-32.72	-18.41
	Site I	-19.41	-42.38	-33.35	-9.04
**DOX-BTZ-2**	Site II	-23.10	-46.07	-44.64	-1.42
	Site III	-30.42	-53.35	-36.94	-16.40

^{a} ΔG^0: Binding energy. ^{b} ΔE_1: Intermolecular interaction energy. ^{c} ΔE_2: Sum of van der Waals energy, hydrogen bonding energy and desolvation free energy. ^{d} ΔE_3: Electrostatic energy.

Upon comparing the binding modes across the three binding sites, it was found that DOX exhibits a stronger preference for binding at site III (Fig. S6). Fig. S6 illustrates that the calculated binding energy for DOX docked at binding site III is -34.56 kJ·mol^{-1}. The consistency between the experimentally determined binding energies and those derived from molecular docking supports the reliability of the findings. The DOX-BSA complex is primarily stabilized in close proximity by hydrophobic interactions and hydrogen bonds (Table S4 and Fig. S6, right). According to the docking simulation, DOX exhibits a preferential affinity for binding site III, where the DOX-BSA complex formation is promoted by a combination of hydrophobic interactions and hydrogen bonds.

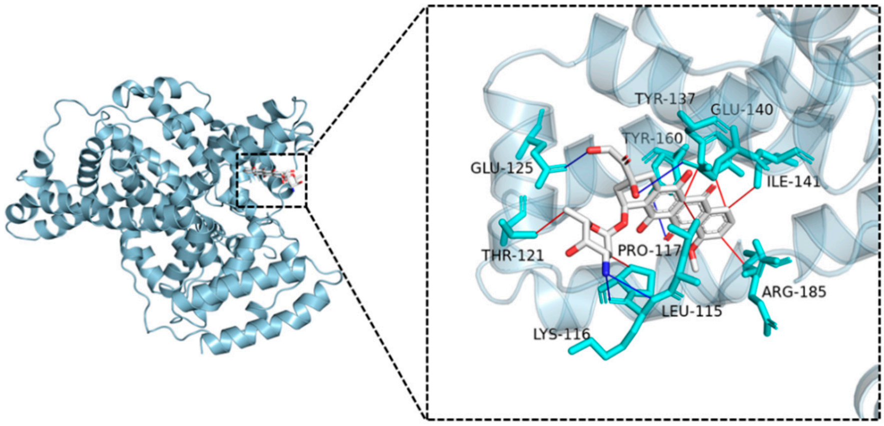

Figure S6. Binding orientations of the lowest docking energy conformation of DOX (rendered in sticks) between binding site III (subdomain IB) of BSA (Left). The enlarged view of the binding locus showing the hydrophobic interaction (red line) and hydrogen bonds (blue line) (Right).

Table S4. Key molecular interactions within the binding pocket.

System	Hydrophobic interactions	Hydrogen bonds	π-Stacking	π-Cation interactions	Metal complexes
**DOX**	Leu-115, Pro-117, Thr-121, Tyr-137, Ile-141, Arg-185	Lys-116, Glu-125, Glu-140, Tyr-160	\	\	\
**DOX-Cu-1**	Lys-114, Leu-115, Pro-117, Tyr-137, Tyr-160, Glu-182	Lys-114, Keu-115, Pro-117, Tyr-160, Leu-178, Glu-182	\	Arg-185	Unk-1
**DOX-Cu-2**	Leu-122, Lys-136, Tyr-137, Tyr-160	Lys-114, Lys-116, Thr-121, Glu-125	Phe-133	\	Unk-1, Glu-125
**DOX-BTZ-1**	His-9, Lys-12	Lys-12, Asp-13, Glu-57, Asp-254, Asp-258	\	Lys-12	\
**DOX-BTZ-2**	Asp-13, Leu-282	Lys-12, Asp-13, Asp-258, Leu-282	\	His-18	\

S4. The preparation of the nanoparticles

S4.1. Optimization of the procedure for the preparation of DOX-Cu@BSA NPs

Table S5. Effect of Water-to-Ethanol Ratio on the formation of DOX-Cu@BSA NPs.

Entry	Water : Ethanol	Particle size (nm)	PDI
**1**	1:2	171.98±4.58	0.12±0.02
**2**	1:2.5	179.31±2.74	0.14±0.03
**3**	1:3	385.93±6.18	0.28±0.01

Table S6. Effect of drop rate of ethanol on the formation of DOX-Cu@BSA NPs.

Entry	Rate ($mL\cdot min^{-1}$)	Particle size (nm)	PDI
**1**	0.5	107.73±0.79	0.20±0.02
**2**	1	172.61±14.9	0.24±0.02
**3**	2	239.32±9.21	0.18±0.04

Table S7. Effect of pH value on the formation of DOX-Cu@BSA NPs.

Entry	pH	Particle size (nm)	PDI
**1**	8	179.94±5.85	0.14±0.01
**2**	9	185.72±15.87	0.22±0.02
**3**	10	217.04±9.43	0.23±0.02

Table S8. Effect of BSA concentration on the formation of DOX-Cu@BSA NPs.

Entry	BSA ($mg\cdot mL^{-1}$)	Particle size (nm)	PDI
**1**	50	3674.05±323	17.24±6.23
**2**	100	182.20±6.45	0.28±0.002
**3**	150	217.50±2.79	0.21±0.01

Table S9. Effect of crosslinking time on the formation of DOX-Cu@BSA NPs.

Entry	Time (h)	Particle size (nm)	PDI
**1**	4	236.39±3.70	0.17±0.04
**2**	6	219.31±5.12	0.23±0.01
**3**	8	170.56±9.58	0.23±0.03

S4.2. Optimization of the procedure for the preparation of DOX-BTZ@BSA NPs

Table S10. Effect of Water-to-Ethanol Ratio on the formation of DOX-BTZ@BSA NPs.

Entry	Water : Ethanol	Particle size (nm)	PDI
1	1:2	191.01±4.2	0.23±0.04
2	1:2.5	168.01±3.79	0.26±0.02
3	1:3	325.54±4.06	0.27±0.01

Table S11. Effect of drop rate of ethanol on the formation of DOX-BTZ@BSA NPs.

Entry	Rate (mL·min^{-1})	Particle size (nm)	PDI
1	0.5	290.31±11.7	0.28±0.03
2	1	188.59±2.38	0.28±0.03
3	2	217.15±4.79	0.27±0.02

Table S12. Effect of pH value on the formation of DOX-BTZ@BSA NPs.

Entry	pH	Particle size (nm)	PDI
1	8	299.54±11.4	0.24±0.01
2	9	134.90±9.34	0.26±0.02
3	10	163.18±6.29	0.24±0.06

Table S13. Effect of BSA concentration on the formation of DOX-BTZ@BSA NPs.

Entry	BSA (mg·mL^{-1})	Particle size (nm)	PDI
1	50	383.43 ±35.1	0.20±0.01
2	100	242.34±2.03	0.29±0.003
3	150	251.53±10.6	0.27±0.03

Table S14. Effect of crosslinking time on the formation of DOX-BTZ@BSA NPs.

Entry	Time (h)	Particle size (nm)	PDI
1	4	222.94±13.6	0.25±0.05
2	6	191.03±7.74	0.29±0.02
3	8	186.79±2.87	0.26±0.03

S5. Cumulative release

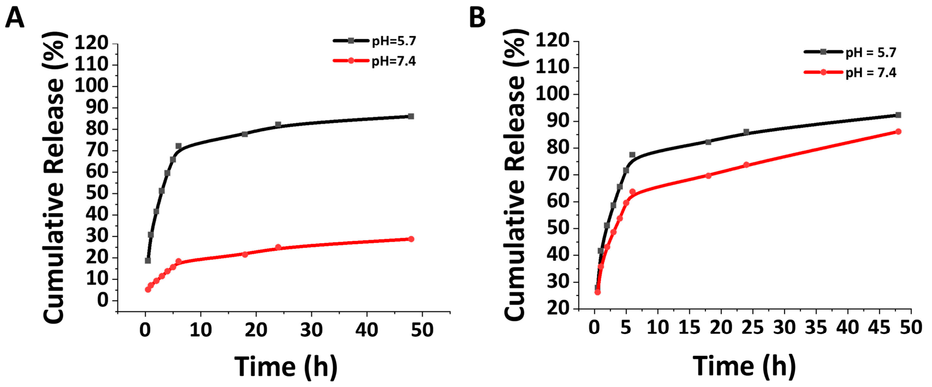

Figure S7. Cumulative release dynamics in PBS across varied conditions (n = 3, independent measurements). (A) DOX-Cu@BSA NPs; (B) DOX-BTZ@BSA NPs.

S6. Cell viability

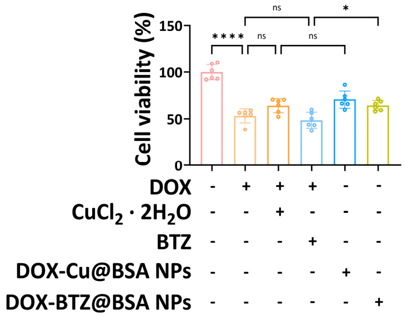

Figure S8. CCK-8 analysis measured cell viability following varied treatments (n = 6, independent experiments). The dosing concentration was fixed at 0.0625 M. **** denotes $p < 0.0001$.
